# Determinants and Prediction of Injury Severities in Multi-Vehicle-Involved Crashes

**DOI:** 10.3390/ijerph18105271

**Published:** 2021-05-15

**Authors:** Xiuguang Song, Rendong Pi, Yu Zhang, Jianqing Wu, Yuhuan Dong, Han Zhang, Xinyuan Zhu

**Affiliations:** 1 School of Qilu Transportation, Shandong University, Jinan 250061, China; songxiuguang@sdu.edu.cn (X.S.); pirendong@mail.sdu.edu.cn (R.P.); 2 Suzhou Research Institute, Shandong University, Suzhou 215123, China; 3 Key Laboratory of Road and Traffic Engineering of the Ministry of Education, Tongji University, Shanghai 201804, China; zhyu@tongji.edu.cn; 4 Shandong High-Speed Group Co. Ltd., Jinan 250002, China; 18119447201@163.com; 5 Shandong High-Speed Construction Management Group Co. Ltd., Jinan 250002, China; 16zhanghan@163.com; 6 Shandong High-Speed Engineering Consulting Group Co. Ltd., Jinan 250061, China; sdgszxy@163.com

**Keywords:** multi-vehicle crash, statistical model, machine learning, unobserved heterogeneity, crash costs

## Abstract

Multi-vehicle (MV) crashes, which can lead to great damages to society, have always been a serious issue for traffic safety. A further understanding of crash severity can help transportation engineers identify the critical reasons and find effective countermeasures to improve transportation safety. However, studies involving methods of machine learning to predict the possibility of injury-severity of MV crashes are rarely seen. Besides that, previous studies have rarely taken temporal stability into consideration in MV crashes. To bridge these knowledge gaps, two kinds of models: random parameters logit model (RPL), with heterogeneities in the means and variances, and Random Forest (RF) were employed in this research to identify the critical contributing factors and to predict the possibility of MV injury-severity. Three-year (2016–2018) MV data from Washington, United States, extracted from the Highway Safety Information System (HSIS), were applied for crash injury-severity analysis. In addition, a series of likelihood ratio tests were conducted for temporal stability between different years. Four indicators were employed to measure the prediction performance of the selected models, and four categories of crash-related characteristics were specifically investigated based on the RPL model. The results showed that the machine learning-based models performed better than the statistical models did when taking the overall accuracy as an evaluation indicator. However, the statistical models had a better prediction performance than the machine learning models had considering crash costs. Temporal instabilities were present between 2016 and 2017 MV data. The effect of significant factors was elaborated based on the RPL model with heterogeneities in the means and variances.

## 1. Introduction

Road traffic injuries have become the eighth-leading cause of death for people of all ages, which remains a serious problem globally. Road traffic injuries are the first cause of death among people 5–29 years of age [1]. Traffic crashes also severely impact social and economic loss [2]. According to the National Highway Traffic Safety Administration (NHTSA), there were 33,654 traffic-related fatalities that involved 51,872 cars in 2018. Single-vehicle (SV) crashes and multi-vehicle (MV) crashes accounted for 56.80% and 43.20%, respectively [3]. However, the number of cars involved in MV and SV crashes accounted for 63.15% and 36.85%, respectively, indicating that MV crashes had more causalities compared to SV crashes. In other words, MV crashes can result in a greater social property loss and cause greater damage to roadside structures and vehicles [2]. As stated above, it is vital to investigate the relationship between crash risk factors and injury-severity in MV crashes. It should be noted that a crash that involves two or more cars is referred to as MV, whereas a crash involving one car is regarded as SV in this study.

In general, existing studies on MV crashes have mainly focused on two categories: critical risk factors analysis and the prediction of injury-severity. As for crash risk factors analysis, many researchers have investigated the relationship between the crash risk factors (i.e., alcohol) and crash injury-severity via statistical models. Binary discrete models, such as the binary logit model or probit model, have widely been used in the studies related to two crash severity levels. Multiple levels of crash severity can be investigated by multinomial models. There have also been many studies that aimed to analyze the unobserved heterogeneity of crash risk factors. Venkataraman et al. [4] employed the random parameter negative binomial model to investigate the heterogeneity in road segments, and the number of vehicles involved in the crash was one of ways to aggregate crashes. The results showed that the heterogeneity could be captured through the random parameters. Seraneeprakarn et al. [5] studied injury-severity in SV and MV crashes that involved at least one hybrid vehicle, and they noted that the estimation model that empowered heterogeneities in the means and variances of random parameters endowed much more flexibility in analyzing the data with the unobserved heterogeneity. Rahimi et al. [6] conducted comprehensive research on the determinants of the injury-severity of truck drivers in single-vehicle truck crashes by developing a random threshold random parameters hierarchical ordered probit model. The increase in probability for fatalities was linked with a wide range of variables (i.e., driver’s education, presence of curves on roadways, and high-speed limit). Shao et al. [7] analyzed the influence of variables related to injury severity in truck-involved rear-end crashes. To analyze data between 2006 and 2015 from the United States for both SV and MV crashes, three random parameters probit models were developed. Specifically, they identified a significant difference between car-strike-truck crashes and truck-strike-car crashes. In another study conducted by Rezapour et al. [8], the differences in SV and MV crashes on downgrades was investigated via the ordered logit model. They identified that there were four significant variables: safety equipment use, lighting conditions, posted speed limit, and lane width, in SV and MV crashes. Hong et al. [2] investigated the impacts of crash risk factors on MV crashes via a double-hurdle approach. By analyzing the data collected from 2011 to 2017 in South Korea, they found that driver violations (i.e., improper distance between vehicles, reversing, and passing) significantly increased the likelihood of injury-severity in MV crashes.

With respect to the prediction of injury-severity, some studies applied machine learning methods to predict the possibility of the injury severity outcomes in recent years.

Support Vector Machine (SVM) has often been used as a prediction tool in traffic-related studies. Li et al. [9] investigated the prediction performance of the SVM model for motor vehicle crashes. It was found that the SVM model was more accurate and effective than the traditional Negative Binomial (NB) model was. Besides, some studies analyzing crash risk factors also used SVM models [10,11]. Random Forest (RF) was also used to predict crash injury-severity. In a study conducted by Harb et al. [12], RF was employed to reveal the associations between crash avoidance maneuvers and crash characteristics (i.e., driver characteristics and vehicle characteristics). It was found that drivers characteristics were the most important factors in all types of crashes. In addition, to achieve a better prediction performance, many researchers employed various kinds of measures to predict crash injury-severity. Sameen and Pradhan [13] used the Recurrent Neural Network (RNN) to predict the injury severity of 1130 crashes collected from 2009 to 2015. Besides that, the back-propagation neural network (BPNN), nearest-neighbor classification (NNC), and K-means clustering were also employed to make predictions about the possibility of injury-severity outcome [14,15].

As summarized above, previous studies have mostly focused on the analysis of the MV crash risk factors. However, limited studies have considered the unobserved heterogeneity and temporal stability on MV crashes. Besides, few studies have predicted the possibility of the injury-severity on MV crashes by using machine learning methods. This was because of the complications that these crashes may have and the potential errors in the model estimations. In this paper, to bridge these gaps in the previous studies, a random parameter logit model with heterogeneities in the means and variances was developed to investigate the crash risk factors in MV crashes. Furthermore, three methods (traditional statistical methods, advanced statistical methods, and machine learning-based methods) dominated the analysis of crash data. To further investigate the differences between advanced statistical models and machine learning-based models, two categories of models were employed to identify the critical contributing factors and to predict the possibility of crash injury-severity.

In the remainder of this paper, we begin by elaborating on statistical and machine learning-based methodologies. A series of likelihood ratio tests on temporal stability are presented. A detailed model evaluation system including four indicators is introduced. The model estimation results and conclusions are then presented, which is followed by the description and processing of data.

## 2. Methodology

In this paper, two kinds of methods, including statistical methods (RPL) and machine learning-based methods (RF), were developed to analyze the critical risk factors and predict the probability of crash injury severities, respectively. This section presents a brief introduction to the above-mentioned methods and the processing of crash data.

### 2.1. Data Processing

The data used in this research were MV crashes collected from HSIS, which provides a large number of major risk factors and outstanding quality of the crash data. The Federal Highway Administration (FHWA) has established this database, which contains 10-state highway safety data since 1987. The three-year (2016–2018) crash data in Washington were utilized in this study. However, as stated in Section 3, only the 2017–2018 crash data were employed after a series of log-likelihood ratio tests.

The investigation of these crashes was based on the “Guidebook for State Data Files California.” According to the variables of “numvehs,” which represents the total number of cars involved in the crash, only crash records involving more than two cars were selected for this study. There were 26,026 MVs reported by the police between 2017 and 2018. After removing the insufficient crash information, 13,478 MVs were left within 2 years (2017–2018). However, each crash record was established on the occupants’ information, indicating that other detailed crash information (i.e., driver characteristics and road characteristics) were doubled, except for the occupants’ information. This can result in a serious collinearity of data. To solve this problem, the related information of the first vehicle involved in MV crashes should be reserved. To be precise, the selection of the first vehicle was based on the “vehno,” which represents the vehicle number.

**Figure 1:**
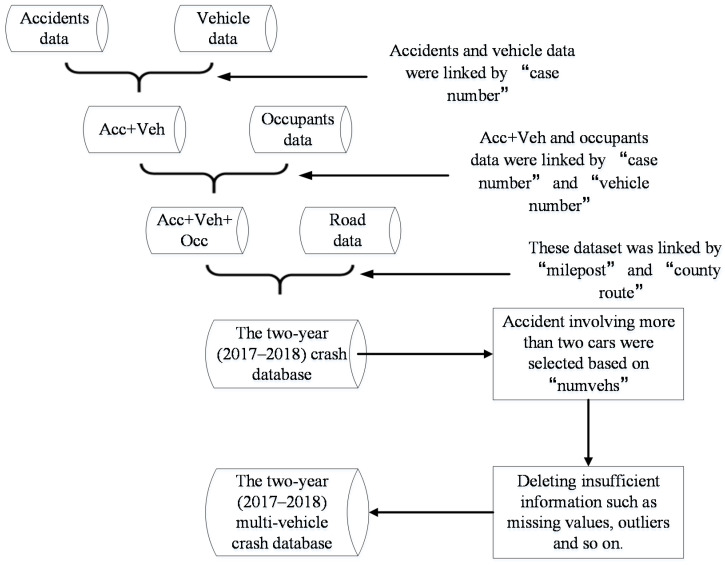
Flowchart of data processing.

The “severity” representing the most severe injury in the crash was employed as the determinant variable. The explanatory variables shown in Appendix A were classified into four categories: driver characteristics, road characteristics, crash characteristics, and occupant characteristics. Factors such as physical condition, helmet, and vehicle violation were found significant on injury-severity level according to some previous research [4,5,6,7,8]. Due to the high account of missing values, they were not considered in this study. However, these indicators can also be represented with other indicators, such as road surface condition, roadway classification, and driver age/gender. More detailed information on data processing is shown in Figure 1.

### 2.2. Random Parameters Logit Model (RPL)

The unobserved heterogeneity originating from various explanatory variables (such as driver characteristics, environmental characteristics, vehicle characteristics, and roadway characteristics) and the energy dissipation via the vehicle structure were crucial in the analysis of crash risk factors. In addition, the resulting effect of energy dissipation varied from occupant physical condition, vehicle safety equipment, and bone mass [5]. Many previous studies emphasized the importance of taking the unobserved heterogeneity into consideration while analyzing crash-related factors. Furthermore, random parameters logit (RPL) models [16,17,18,19], random parameters logit models with heterogeneities in the means [20,21], and random parameters logit models with heterogeneities in the means and variances [5,22,23] have all been successfully utilized in the investigation of crash injury-severity. Hence, to account for the unobserved heterogeneity, the random parameters logit model with heterogeneities in the means and variances was used in this study.

To achieve a random parameter logit model, an injury-severity propensity function $V_{ki}$ was defined, as shown in Equation (1):

(1) $$V_{ki}=X_{ki}\beta_{k}+\varepsilon_{ki}$$

where $V_{ki}$ determines the probability of injury-severity category *k* (property damage only, possible injury, and fatal) in crashes *i*, and $X_{ki}$ is a vector of explanatory variables (driver characteristics, road characteristics, crash characteristics, and occupant characteristics) and presents a vector of the estimable parameters for injury-severity level *k*. Given the crash-specific unobserved heterogeneity, the estimable parameters $\beta_{k}$ are allowed to vary from different observations through a density function, where *φ* represents a vector of parameters of the density function. In addition, $\varepsilon_{ki}$ is a random term that follows a type I extreme value (i.e., Gumbel) distribution. Thus, the calculation of the probability for each crash injury-severity level is shown in Equation (2) [24]:

(2) $$P_{i}\left(k\right)=\int \frac{e^{X_{ki}\beta_{k}}}{\sum_{i=1}^{n}e^{X_{ki}\beta_{k}}}f\left(\beta |\varphi \right)d\beta$$

where $P_{i}\left(k\right)$ represents the possibility of crash *i* with injury-severity level *k*. Furthermore, the random parameters with heterogeneities in the means and variances is defined as Equation (3) by Seraneeprakarn et al. [5].

(3) $$\beta_{k}=\bar{\beta }+\delta_{k}Z_{k}+\sigma_{k}\exp(\omega_{k}W_{k})\upsilon_{k}$$

where $\bar{\beta }$ represents the mean estimated parameters across all crashes, $Z_{k}$ is a vector of crash-related variables capturing the heterogeneity in the means for all crashes, and $\delta_{k}$ is the corresponding vector of estimated parameters. $W_{k}$ is a vector of crash-specific variables that explain the heterogeneity in the standard deviation $\sigma_{k}$ with corresponding parameter vector $\omega_{k}$, and $\upsilon_{k}$ is a disturbance term.

### 2.3. Random Forest

Random Forest (RF), Support Vector Machine (SVM), and Back-Propagation Neural Network (BPNN) had all been widely and successfully used in predicting the possibility of injury-severity outcome [25,26,27,28]. Choosing a suitable method for the crash prediction is critical. In addition, the crash datasets are high-dimensional, imbalanced, and have a large sample size. The determinant variable was divided into three categories. SVM cannot handle well datasets with a large sample size. A large sample size would take a long computational time. Besides, SVM is good at binary classification rather than multi-classification. The Back-Propagation Neural Network (BPNN) would also take a long training time, and the structure of the neural network is often determined based on experience. It restricts the generalization of the model. Furthermore, the performance of BPNN has a strong dependence on the sample. Imbalanced datasets are not conducive to the prediction of BPNN. However, the RF is good at dealing with high-dimensional datasets and would take less computational time than others at the same sample size. In addition, RF considers the interactive influence between each feature during the training. This makes the prediction results more reliable. Therefore, the RF was utilized in this paper to further validate and evaluate its ability in the performance of crash prediction.

**Figure 2:**
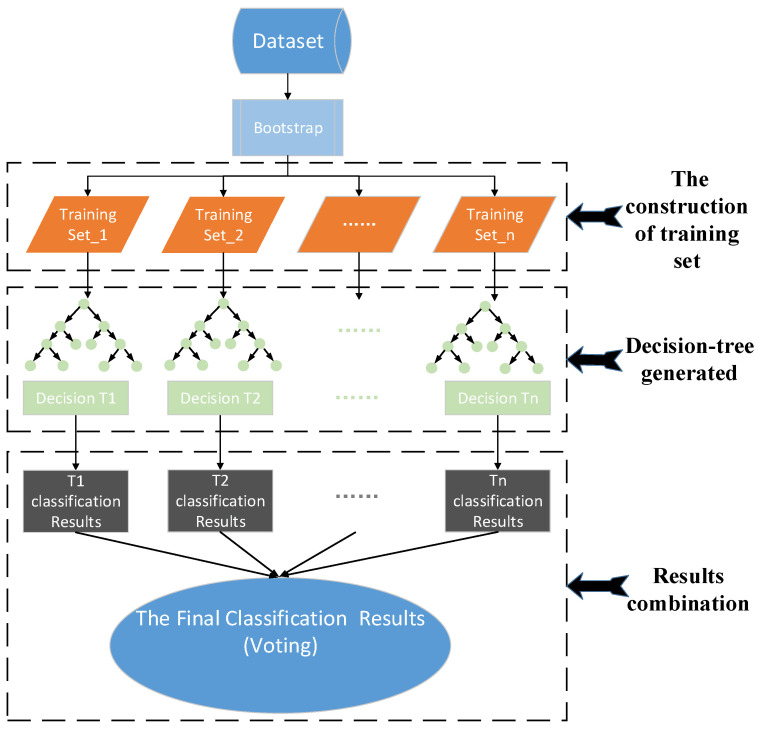
The construction of Random Forest (RF) model.

Random Forest (RF) is an ensemble learning method. RF is based on bagging by taking decision trees as the base learner, which was proposed by Breiman in 2001 [29]. Given the input dataset represented as (*xi*,*yi*), *xi* represents all the crash-related explanatory variables, and *yi* is the injury severity. The construction of the RF model is shown in Figure 2, which consists of three parts:

(a) The construction of a training set. The training set is extracted from the original dataset by using bootstrap. Additionally, the bootstrap is a kind of nonparametric Monte Carlo method, ensuring each sample has the same chance to be selected.

(b) Decision-tree generation. Each decision-tree is generated by part of all features, which is randomly selected. That is, each decision-tree is a base learner.

(c) Results combination. Each training set will be classified by their own decision-tree. Then, the final classification result is the mode value in the all-decision-tree prediction result. In addition, each decision-tree result has the same weight in the final vote.

### 2.4. Model Evaluation

According to previous studies [13,30], a test dataset was used to evaluate the prediction performance of different models, and the prediction results for each model can be described by a confusion matrix (also called an error matrix) [31], as shown in Table 1. To make full use of the crash datasets and to perform a robust evaluation, a 10-fold cross-validation was used in this paper for evaluating the predictive performance of the RF model [32].

In this error matrix, the column values are the predicted results while the row values are the actual results. That is, *P_ij_* is defined as the number of crashes with injury-severity level *i*, but it is predicted as *j*. *R_ij_* is the ratio of the prediction result over the number of crashes with injury-severity level *j*. Furthermore, the overall correct prediction ratio is calculated as Equation (6).

(4) $$R_{\mathrm{overall}}=\frac{\sum_{i=1}^{s}p_{ii}}{\sum_{i=1}^{s}N_{i}}$$

Additionally, for each injury-severity level *i*, the calculated value *R_ii_* can indicate the correct prediction ratio.

However, under particularly crash-related conditions (weather, vehicle type, driver age, etc.), the indicator $R_{\mathrm{overall}}$ may not provide reliable results. There are two reasons: (a) A large account of crashes are from one specific severity level, such as Property Damage Only (PDO), which accounted for a large portion in many crash datasets. An insensitive prediction model would regard all the crashes as a specific frequent severity level, which would result in a higher $R_{\mathrm{overall}}$. However, a sensitive model would regard the specific frequent severity level as the same as the other severity levels, which may have a lower $R_{\mathrm{overall}}$; and (b) $R_{\mathrm{overall}}$ indicated that the value of each injury-severity level was equal. That is, the social influence and property loss caused by the different injury-severity levels were equal [14].

Hence, in this study, another three indicators aiming to evaluate the prediction accuracy were used. These indicators took crash-related economic costs in the evaluation of prediction performance, as can be shown in Equation (7).

(5) $${CCC}_{i}={ECC}_{i}+{QALYCC}_{i}$$

The comprehensive crash cost (*CCC*) consisted of two parts: economical crash costs (*ECC*) and quality-adjusted life years (*QALY*). It is worth explaining the meaning of *QALY* to gain a further understanding of Equation (7). *QALY* is an indicator that can estimate the value of the lost quality-of-life due to crashes by quantifying the value of some behaviors people would take to avoid injury or death [33]. That is, *ECC* and *QALY* represent observable and unobservable costs due to crashes, respectively [34].

Moreover, the comprehensive crash cost of 2017, as shown in Table 2, was updated by using the consumer price index (*CPI*) and median usual weekly earnings (*MUWE*) based on Crash Costs for Highway Safety Analysis (*CCHSA*) [33].

Based on the comprehensive crash cost of 2017, the actual overall costs of crashes (*AOCC*) and the predicted overall costs of crashes (*POCC*) were defined as Equations (8) and (9), respectively.

(6) $$AOCC=\sum_{i=1}^{j}N_{i}CCC_{i}$$

(7) $$POCC=\sum_{i=1}^{s}\sum_{i=1}^{s}p_{ij}CCC_{j}$$

Furthermore, the overall prediction mean absolute error (*OPMAE*), overall prediction absolute percentage error (*OPAPE*), and overall prediction root-mean-squared error (*OPRMSE*) were defined as Equations (10)–(12), respectively. Both *OPMAE* and *OPRMSE* can evaluate the absolute errors between the predicted and actual costs. The *OPAPE* was used to measure the relative errors between them.

(8) $$OPMAE=\frac{\left|AOCC-POCC\right|}{N}$$

(9) $$OPAPE=\frac{\left|AOCC-POCC\right|}{AOCC}\times 100\%$$

(10) $$OPRMSE=\sqrt{\frac{{\left(AOCC-POCC\right)}^{2}}{N}}$$

## 3. Results and Discussion

In this section, likelihood ratio tests of temporal stability were conducted, and the prediction performance of the two selected models was comprehensively measured. First, two series of likelihood ratio tests were conducted to examine the temporal stability of three-year crash datasets. Secondly, the process of determining various parameters for machine learning-based models was elaborated in detail. Thirdly, the prediction performance among these models was evaluated by comparing four indicators (*R*_overall_, *OPMAE*, *OPAPE*, and *OPRMSE*). The final step was to analyze the effects of significant variables on injury-severity.

### 3.1. Likelihood Ratio Tests

Many previous studies found that the critical factors of crash injury severity showed temporal instability [35]. Considering this, a series of tests were conducted in this paper to examine the differences between different years’ MV crashes via likelihood ratio tests.

To comprehensively examine the temporal stability of MV crashes injury severity, two series of likelihood ratio tests were conducted. The first series of likelihood ratio tests were utilized to identify whether the different estimated parameters were stable between two individual years. This likelihood ratio test is defined as [35,36]:

(11) $$\chi_{t1}^{2}=-2[LL(\beta_{y1y2})-LL(\beta_{y1})]$$

where $LL(\beta_{y1y2})$ is the log-likelihood at convergence for the model estimating parameters from *y2* while using data subset *y1*, whereas $LL(\beta_{y1})$ denotes the log-likelihood at convergence for the model using data from *y1*’s data. For each model comparison, the test was carried out the other way around based on the *y1* subset and *y2* subset to obtain two different results. That is, taking the estimated parameters of the 2017 model as the starting values and employing them in the 2016 data, the $\chi^{2}$ between 2017 and 2016 can be calculated. The calculated $\chi^{2}$ was 35.06 with 9 freedoms in this dataset, illustrating that the null hypothesis that the 2017 and 2016 data are the same can be rejected at a high confidence level (the corresponding confidence level more than 99.99%).

Likewise, other two-year periods can be identified as equal to the null hypotheses being rejected at a high confidence level except the 2017 and 2018 data. Table 3 shows $\chi^{2}$ values with degrees of freedom in parentheses and confidence level in square brackets. Cells in italics indicate the null hypothesis that the temporal stability cannot be rejected at a high confidence level (>95%).

Besides, for investigating the temporal stability between the joint model and each separate model, the second series of likelihood ratio tests can be written as [35]:

(12) $$\chi_{t2}^{2}=-2[LL(\beta_{2017–2018})-\sum_{2017}^{2018}LL(\beta_{i})]$$

where $LL(\beta_{2017–2018})$ identifies the log-likelihood at the convergence of the model corresponding to all available year data (2017 and 2018), and $LL(\beta_{i})$ represents the log-likelihood at convergence of the model with only one year (2017 or 2018) data. The value of $\chi^{2}$ was 1.62 with 14 degrees of freedom (the corresponding confidence level was about 0.00%). This result showed that the null hypothesis that the contributing factors of crash injury severity in separate models is of temporal stability cannot be rejected at a high confidence level. The estimated parameters of the joint model can be utilized for analysis.

### 3.2. Model Estimation

**Figure 3:**
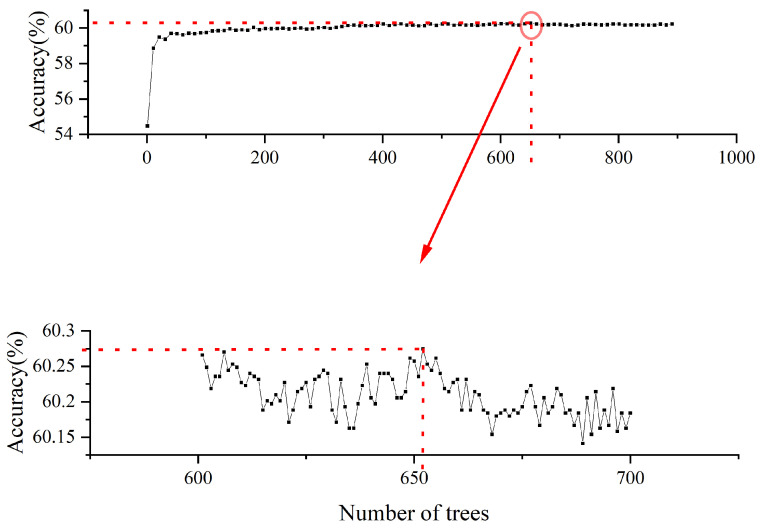
The learning curve of the RF model.

The prediction accuracy with the number of trees was evaluated using the learning curve, which aimed to find the best prediction accuracy. As shown in Figure 3, the prediction accuracy reached its maximum value when the number of trees was 652, which indicated that the RF model constructing 652 trees obtained the best prediction performance. According to previous research, the number of optimal features used for splitting was to be set as the square root of all features.

### 3.3. Model Prediction and Discussion

As shown in Table 4 the *OPAPE* of the statistical models was 3.61%, while the machine learning-based model was 27.12%. In the comparison of these two methods, the *OPAPE* values of the statistical model were lower than those of the machine learning-based model. That is, the statistical model had a better performance concerning crash costs than the machine learning model had. However, the overall prediction accuracy of the machine learning-based model was higher than that of the statistical model (the $R_{\mathrm{overall}}$ of the statistical model was 56.59%, and the $R_{\mathrm{overall}}$ of the machine learning-based method was 67.16%). Furthermore, the statistical and machine learning-based models had a similar trend, respectively, regarding *OPMAE* and *OPRMSE*.

The interpretation of the indicators shown in Table 4 depended on the way in which to utilize them in practice. For example, as a road designer, the relationship between different conditions (i.e., road characteristics and environmental characteristics) with the potential injuries is crucial. Therefore, the prediction models were selected based on the overall prediction accuracy. As for insurance companies, the crash costs deserve more attention. Hence, the crash-costs-related indicators such as *OPMAE*, *OPAPE*, and *OPRMSE* should be selected first. It was easy to conclude that the statistical methods had a better performance than the machine learning-based models considering crash costs.

Additionally, a higher overall prediction accuracy did not imply a better prediction performance on a specific type of injury-severity, due to the different account of various injury severities. In order to choose one model that can predict a specific level of injury-severity as accurate as possible, the *R_ii_* indicator in the confusion matrix should be selected. As shown in Table 5, the machine learning-based model achieved the best prediction performance (its *R_ii_* was 85.14%) when the property damage only was the only concern. As for injury, the prediction accuracy of the statistical model reached 34.03%.

### 3.4. Effect of Significant Factors

After analyzing the prediction performance among the selected models, a detailed investigation of the effects of significant crash-related factors was critical to further understand these models. Depending on the mathematical definition of these models (statistical/machine learning-based models), statistical models were able to clearly explain the effects of various parameters by utilizing coefficients and significance. Furthermore, the RPL model was estimated by the simulated maximum likelihood, which was an efficient method to random draws. To estimate more accurate parameters, 500 Halton draws were used in this study. The density function f(β|φ) followed a normal distribution, similar to previous studies [22,23,35]. The whole estimated results are shown in Appendix B.

Regarding the driver characteristics, among the characteristics of drivers, “old-aged driver” was found to significantly decrease the likelihood of property damage only. “Middle-aged driver” increased the likelihood of property damage only. “Male driver” was found to increase the likelihood of property damage only, which was in line with previous studies [23]. In addition, taking “sudden slowing maneuvers” can significantly decrease the possibility of Fatal injury compared with “skipping involved.”

The roadway characteristics found to be significant were: “Wet/snow/slush/ice road surface” and “Rural freeways.” “Wet/snow/slush/ice road surface” increased the likelihood of property damage only. The indicator “Rural freeways” was found to be statistically significant in increasing the likelihood of the Fatal injury.

Appendix B also shows that the “crash not occurring at intersection or driveway” increased the possibility of the property damage only. The indicator “weekend” decreased the likelihood of Injury.

With regard to the characteristics of the occupant, “Male occupant” was found to decrease the likelihood of the property damage only. The indicator “Old-aged occupant” was found to decrease the likelihood of the property damage only in our study, which was in line with some previous studies [23]. The indicator “Ejected” was found to significantly decrease the likelihood of the property damage only. In addition, the indicator “second row” was found to decrease the likelihood of the Injury.

As indicated in Appendix B, two variables were identified as random parameters in this study: “Occupant restraints” and “Male driver.” The indicator of “Occupant restraints” followed a normal distribution with a mean of −1.5017 and a standard deviation of 4.5840, which means that this variable was negative for observations of 62.84% (decreasing the likelihood of the injury) and positive for observations of 37.16% (increasing the likelihood of the injury). That is, the crashes were not prone to occur when the occupants’ safety equipment was used. As for the analysis of “Male driver”, it can also be interpreted through the same way.

**Figure 4:**
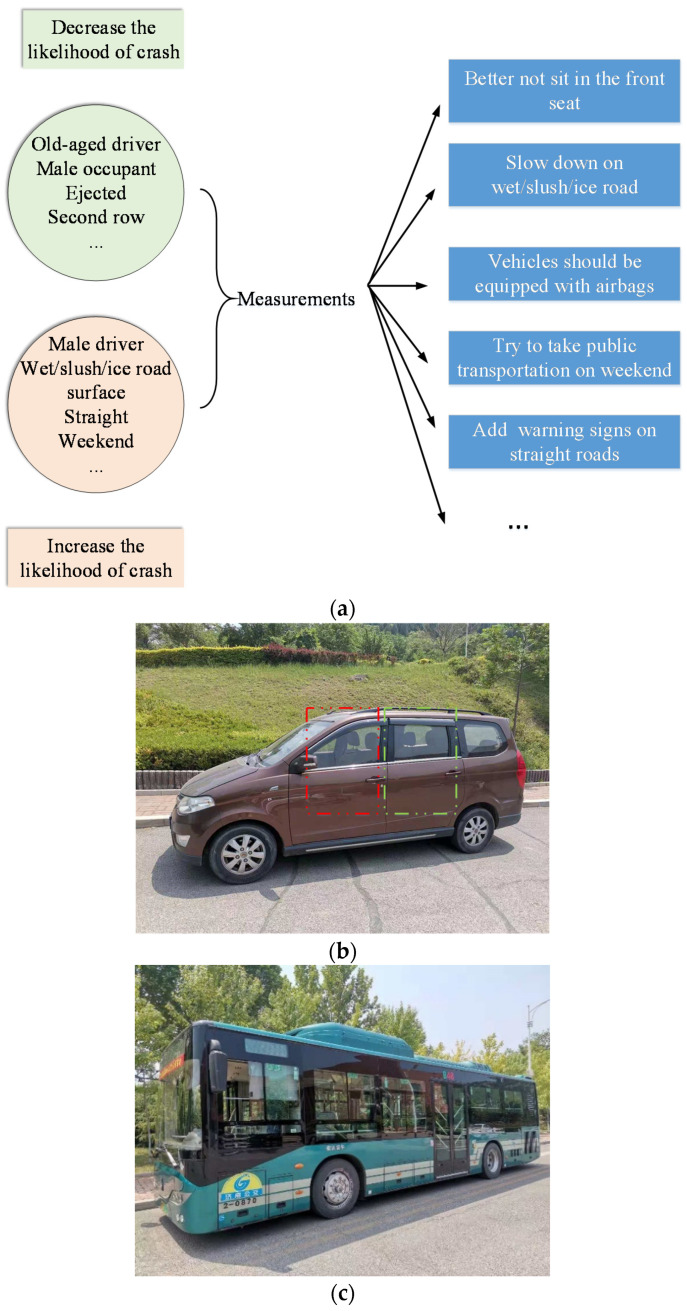
The measurements for road safety. (**a**) The relationship between crash-related factors and measurements, (**b**) better not sit in the front seat, and (**c**) try to take public transportation on weekends.

With respect to the random parameters with heterogeneity in the means, the indicator of occupant restraints [I] and Male driver [I] were found to produce random parameters with heterogeneity in the means. The negative values of −0.5543 indicated that the mean of occupant restraints indicator decreased if the driver took “sudden slowing maneuvers,” also meaning that the possibility of injury was decreased. As for the heterogeneity in the variance of random parameters, the “Middle-aged driver” was found to decrease the variance of the indicator “Occupant restraints.” Based on the above analysis, the corresponding measurements decreasing the likelihood of crashes is shown in Figure 4.

## 4. Conclusions

This research employed two methods (statistical methods: RPL; machine learning-based method: RF) to analyze significant crash-related factors and to predict the possibility of injury-severity outcomes based on the dataset of 13,667 crashes extracted from the HSIS database.

As for the crash prediction, the overall accuracies of the RF and RPL model were 56.59% and 67.14%, respectively. The *OPMAE* and *OPAPE* of these two models were 2143 and 3.61%, and 14,076 and 27.12%, respectively. Regarding crash costs, the *OPRMSE* of the RPL and RF model were USD 137 and USD 895 (millions).

For significant crash-related factors, the variables “old-aged driver,” “Male occupant,” “Old-aged occupant,” “Ejected,” and “Second row” may decrease the likelihood of crash injury severity; while variables “Male driver,” “Wet/snow/slush/ice road surface,” “Straight,” “Not at intersection or driveway,” and “Weekend” could increase the possibility of crash injury severity. In addition, the indicator “Occupant restraints” and “Male driver” were identified as random parameters. The above findings could be applied by various walks of life (e.g., the government, transportation-related enterprise, and insurance company) to improve transportation safety and reduce the crash costs.

It should be noted that there are still some limitations in this study. Many prevailing machine learning-based methods, such as ANN and KNN, were not used for comparison in this study. The imbalance datasets may result in biases in crash prediction and critical risk factors analysis. Besides that, the out-of-date crash datasets may result in some bias in the analysis of crash critical factors, and some factors (road alignment, occupant/driver physical condition, etc.) were not considered in this research. Future studies will focus on these above-mentioned issues.

## Figures and Tables

**Table 1:** Confusion matrix (error matrix) of an injury-severity level prediction model.

	Predicted	Injury-Severity Level			Actual Number of Crashes
Actual		Property Damage Only (PDO)	Injury (I)	Fatal Injury (FI)	
Injury-Severity Level	Property Damage Only (PDO)	*P_11_*	*P_12_*	*P_13_*	*N_1_*
		*R_11_*	*R_12_*	*R_13_*	
	Injury (I)	*P_21_*	*P_22_*	*P_23_*	*N_2_*
		*R_21_*	*R_22_*	*R_23_*	
	Fatal Injury (FI)	*P_31_*	*P_32_*	*P_33_*	*N_3_*
		*R_31_*	*R_32_*	*R_33_*	

Note: *P*, the prediction result; *R*, the ration of the prediction result over the number of crashes; *N*, actual number of crashes.

**Table 2:** 2017 comprehensive crash unit cost based on injury-severity level (USD).

Injury-Severity Level	Economic Crash Costs	QALY Crash Unit Costs	Comprehensive Crash Unit Cost
Property Damage Only (PDO)	12,456	0	12,456
Injury (I)	46,132	97,535	143,667
Fatal Injury (FI)	588,738	3,173,900	3,762,638

Note: QALY, quality-adjusted life years.

**Table 3:** Likelihood ratio test results between different years.

	2016	2017	2018
2016	-	35.06 (9) [>99.99%]	32.02 (10) [>99.96%]
2017	7.48 (13) [*12.42%*]	-	3.56 (10) [*3.49%*]
2018	8.94 (13) [*22.25%*]	2.84 (9) [*2.97%*]	-

**Table 4:** Comparison of model prediction based on four indicators.

Methods	Statistical Methods	Machine Learning Methods
	RPL	RF
*R*_overall_	56.59%	67.16%
*OPMAE*	2143	14,076
*OPAPE*	3.61%	27.12%
*OPRMSE* (USD millions)	137	895
*POCC* (USD millions)	252	153
*AOCC* (USD millions)	243	209

Note: *R*_overall_, the overall correct prediction ratio; *OPMAE*, the overall prediction mean absolute error; *OPAPE*, the overall prediction absolute percentage error; *OPRMSE*, the overall prediction root-mean-squared error; *POCC*, the predicted overall costs of crashes; *AOCC*, the actual overall costs of crashes.

**Table 5:** Error matrix for injury-severity prediction model.

Injury-Severity Level	Method	Property Damage Only (PDO)	Injury (I)	Fatal (F)
Property Damage Only (PDO)	RPL	1881	918	3
		67.13%	32.76%	0.11%
	RF	2408	419	1
		85.14%	14.81%	0.03%
Injury (I)	RPL	850	440	3
		65.74%	34.03%	0.23%
	RF	909	306	1
		74.75%	25.16%	0.08%
Fatal (F)	RPL	4	2	0
		66.67%	33.33%	0%
	RF	0	0	0
		0%	0%	0%

Note: RPL, random parameters logit model with heterogeneity in means and variances; RF, random forest model.

## Data Availability

The data that support the findings of this study are available from the corresponding author, upon reasonable request.

## Appendix A

**Table:**

Explanatory Variable	PDO		Injury		Fatal		Total	
**Total crashes**	9287	68.90%	4180	31.01%	11	0.08%	13,478	
**Driver characteristics**								
Driver gender								
Male driver	5512	40.90%	2280	16.92%	5	0.04%	7797	57.85%
Female driver	3775	28.01%	1900	14.10%	6	0.04%	5681	42.15%
Driver age								
Young driver	1458	10.82%	628	4.66%	3	0.02%	2089	15.50%
Middle-aged driver	6718	49.84%	2951	21.89%	5	0.04%	9674	71.78%
Elder driver	1111	8.24%	601	4.46%	3	0.02%	1715	12.72%
Driver restraint								
No restraints used	33	0.24%	19	0.14%	3	0.02%	55	0.41%
Lap belt/shoulder or other restraints used	9254	68.66%	4161	30.87%	8	0.06%	13,423	99.59%
Driver mistake action								
Skidding involved	175	1.30%	60	0.45%	2	0.01%	237	1.76%
Avoiding maneuvers	156	1.16%	47	0.35%	0	0.00%	203	1.51%
Sudden slowing maneuvers	4089	30.34%	1505	11.17%	1	0.01%	5595	41.51%
Stopped vehicle	4145	30.75%	2061	15.29%	2	0.01%	6208	46.06%
**Vehicle characteristics**								
Carry hazardous material								
Yes	0	0.01%	2	0.04%	4	0.00%	6	0.04%
No	5417	40.21%	8055	59.79%	0	0.00%	13,472	99.96%
**Road characteristics**								
Roadway classification								
Urban freeways	5463	40.53%	2337	17.34%	3	0.02%	7803	57.89%
Urban multilane roads	2607	19.34%	1272	9.44%	0	0.00%	3879	28.78%
Rural freeways	562	4.17%	232	1.72%	3	0.02%	797	5.91%
Rural multilane roads	655	4.86%	339	2.52%	5	0.04%	999	7.41%
Road characteristics								
Straight	8589	63.73%	3845	28.53%	10	0.07%	12,444	92.33%
Curve	698	5.18%	335	2.49%	1	0.01%	1034	7.67%
Federal function class								
Rural collector	1221	9.06%	573	4.25%	8	0.06%	1802	13.37%
Urban collector	8066	59.85%	3607	26.76%	3	0.02%	11,676	86.63%
Road surface type								
Portland concrete cement	2440	18.10%	1006	7.46%	0	0.00%	3446	25.57%
Asphalt concrete	6847	50.80%	3171	23.53%	11	0.08%	10,029	74.41%
Brick/gravel/dirt	0	0.00%	3	0.02%	0	0.00%	3	0.02%
**Crash characteristics**								
Day of week								
Non-weekend	6038	44.80%	2796	20.74%	8	0.06%	8842	65.60%
Weekend	3249	24.11%	1384	10.27%	3	0.02%	4636	34.40%
Location of the crash								
Intersection-related	2316	17.18%	1135	8.42%	3	0.02%	3454	25.63%
Driveway-related	279	2.07%	177	1.31%	1	0.01%	457	3.39%
Not at intersection or driveway	6692	49.65%	2868	21.28%	7	0.05%	9567	70.98%
Weather								
Clear	7354	54.56%	3410	25.30%	5	0.04%	10,769	79.90%
Cloudy	1689	12.53%	657	4.87%	4	0.03%	2350	17.44%
Raining/snowing	154	1.14%	69	0.51%	0	0.00%	223	1.65%
Fog/wind/other	90	0.67%	44	0.33%	2	0.01%	136	1.00%
Light condition								
Daylight	7233	53.67%	3261	24.19%	7	0.05%	10,501	77.91%
Dusk-dawn	319	2.37%	137	1.02%	0	0.00%	456	3.38%
Dark, light on	1274	9.45%	594	4.41%	2	0.01%	1870	13.87%
Dark, light off	461	3.42%	188	1.39%	2	0.01%	651	4.83%
Roadway surface								
Dry	6722	49.87%	3104	23.03%	4	0.03%	9830	72.93%
Wet/snow/slush/ice	2538	18.83%	1060	7.86%	7	0.05%	3605	26.75%
Other	27	0.20%	16	0.12%	0	0.00%	43	0.32%
**Occupant characteristics**								
Age								
Young passenger	5019	37.24%	1951	14.48%	4	0.03%	6974	51.74%
Middle-aged passenger	3352	24.87%	1654	12.27%	5	0.04%	5011	37.18%
Elder passenger	916	6.80%	575	4.27%	2	0.01%	1493	11.08%
Gender								
Male	4115	30.53%	1572	11.66%	5	0.04%	5692	42.23%
Female	5172	38.37%	2608	19.35%	6	0.04%	7786	57.77%
Seat position								
First row	4934	36.61%	2499	18.54%	6	0.04%	7439	55.19%
Second row	1237	9.18%	446	3.31%	0	0.00%	1683	12.49%
Third row	3116	23.12%	1235	9.16%	5	0.04%	4356	32.32%
Eject								
Not ejected	9281	68.86%	4175	30.98%	7	0.05%	13,463	99.89%
Ejected	6	0.04%	5	0.04%	4	0.03%	15	0.11%
Occupant Restraint								
No restraints used	34	0.25%	33	0.24%	3	0.02%	70	0.52%
Lap belt/shoulder or other used	9253	68.65%	4147	30.77%	8	0.06%	13,408	99.48%

## Appendix B

**Table:**

Variable	Random Parameters Logit Model (with Heterogeneity in Means and Variances)	
	Parameters Estimate	z-Stat
Constant (PDO)	7.0652	15.68
Constant (I)	5.4921	11.68
Driver characteristics		
Old-aged driver (1 if driver is older than 60 years old; 0 otherwise) (PDO)	−1.3907	−3.66
Middle-aged driver (1 if driver is between 25 and 60 years old; 0 otherwise) (PDO)	1.4329	3.34
Male driver (1 if the gender of driver is male; 0 otherwise) (PDO)	0.7133	−3.44
Sudden slowing maneuvers (1 if the Driver mistake action is Sudden slowing maneuvers; 0 otherwise) (FI)	−2.0871	−1.68
Road characteristics		
Wet/snow/slush/ice road surface (1 if the road surface is wet/snow/slush/ice; 0 otherwise) (PDO)	0.2841	2.09
Rural freeways (1 if the road classification is rural freeways; 0 otherwise) (F)	1.8023	2.21
Crash characteristics		
Not at intersection or driveway (1 if the crash occurred not at intersection or driveway; 0 otherwise) (PDO)	0.2232	1.73
Weekend (1 if weekend; 0 otherwise) (I)	−0.1791	−1.56
Occupant characteristics		
Male occupant (1 if the gender of occupant is male; 0 otherwise) (PDO)	−0.5782	−2.39
Old-aged occupant (1 if occupant is older than 60 years old; 0 otherwise) (PDO)	−0.8212	−2.30
Ejected (1 if occupant is ejected; 0 otherwise) (PDO)	−4.2151	−4.30
Second row (1 if the occupant seated in second row; 0 otherwise) (I)	−0.4940	−2.29
Random parameters		
Occupant restraints (1 if occupant’s safety equipment is used; 0 otherwise) (I)	−1.5017	−2.56
Standard deviation of “Occupant restraints” (I)	4.5840	3.45
Male driver (1 if the gender of driver male; 0 otherwise) (I)	0.6905	2.38
Standard deviation of “Male driver” (I)	3.1585	2.66
Heterogeneity in the mean of the random parameters		
Occupant restraints (I): Sudden slowing maneuvers	−0.5543	−2.83
Male driver (I): Sudden slowing maneuvers	−0.8786	−2.54
Heterogeneity in the variances of the random parameters		
Occupant restraints (I): Middle-aged driver	−0.4272	−2.19
Model statistics	-	-
Number of observations	13,478	-
AIC	16,593	-
BIC	16,743	-
McFadden $\rho^{2}$	0.44	-

PDO, Property Damage Only; I, Injury; FI, Fatal Injury.
